# Care use and its intensity in children with complex problems are related to varying child and family factors: A follow-up study

**DOI:** 10.1371/journal.pone.0231620

**Published:** 2020-05-06

**Authors:** Noortje M. Pannebakker, Paul L. Kocken, Paula van Dommelen, Krista van Mourik, Ria Reis, Sijmen A. Reijneveld, Mattijs E. Numans

**Affiliations:** 1 Department of Child Health, TNO, Leiden, Netherlands; 2 Department of Public Health and Primary Care, Leiden University Medical Center, Leiden, Netherlands; 3 Department of Health Sciences, University of Groningen, University Medical Center Groningen, Groningen, Netherlands; 4 Department of Public Health and Primary Care and LUMC Campus The Hague, The Hague, Netherlands; International Telematic University Uninettuno, ITALY

## Abstract

**Background:**

There is little evidence on the child and family factors that affect the intensity of care use by children with complex problems. We therefore wished to identify changes in these factors associated with changes in care service use and its intensity, for care use in general and psychosocial care in particular.

**Methods:**

Parents of 272 children with problems in several life domains completed questionnaires at baseline (response 69.1%) and after 12 months. Negative binominal Hurdle analyses enabled us to distinguish between using care services (yes/ no) and its intensity, i.e. number of contacts when using care.

**Results:**

Change in care use was more likely if the burden of adverse life events (ALE) decreased (odds ratio, OR = 0.94, 95% confidence interval, CI = 0.90–0.99) and if parenting concerns increased (OR = 1.29, CI = 1.11–1.51). Psychosocial care use became more likely for school-age children (vs. pre-school) (OR = 1.99, CI = 1.09–3.63) if ALE decreased (OR = 0.93, CI = 0.89–0.97) and if parenting concerns increased (OR = 1.26, CI = 1.10–1.45). Intensity of use (>0 contacts) of any care decreased when ALE decreased (relative risk, RR = 0.95, CI = 0.92–0.98) and when psychosocial problems became less severe (RR = 0.38, CI = 0.20–0.73). Intensity of psychosocial care also decreased when severe psychosocial problems became less severe (RR = 0.39, CI = 0.18–0.84).

**Conclusions:**

Changes in care-service use (vs. no use) and its intensity (>0 contacts) are explained by background characteristics and changes in a child’s problems. Care use is related to factors other than changes in its intensity, indicating that care use and its intensity have different drivers. ALE in particular contribute to intensity of any care use.

## Introduction

Little research has been conducted on factors affecting the intensity of care use by children with complex problems (CP*)*. *The need of these* children for health *and social* services, especially psychosocial care, is typically greater than would be expected *based on* their chronic physical, developmental, behavioral or emotional conditions; *this is the case because their problems interact and enhance vulnerabilities* [1–4]. *These children are also referred to as members of troubled families or hotspotters [5–7]. Children with complex problems form the top 5% of children with the most challenging problems, amounting in the Netherlands to 170,000 children. Western countries struggle to organize effective and efficient care pathways for these children [8]. As a result, a major part of the budgets of psychosocial services is spent on children with CP [9,10].*

*The determinants of care use as such have been studied in depth, revealing several factors that impact access [11–16]. Less attention was paid to understanding the intensity of care use*, i.e. the number of contacts with care providers. The scarce literature shows that higher intensity of care-service use by children with CP is related to two main groups of factors: child factors (age and impact of psychosocial problems); and parental factors (educational level, healthcare use, social support, and parental psychosocial problems) [17–20]. *Research shows that the determinants affecting care use (yes/no) and the intensity with which it is used differ when studied simultaneously* [17–19]. *This suggests that intensity of care use may be a unique component of the help-seeking behavior of families with a child with complex problems*. *A better understanding of the intensity of care use will help us to organize more efficient care paths for these children*.

Research on determinants of care use is often guided by Andersen and Newman’s behavioral-health model [21]. *This model was developed to explain the use of care by an individual or population and has shown its value as comprehensive model for this purpose in health care research during the past decades [22,23].* The model describes care use on the basis of three factors: 1. predisposing factors, i.e., a child’s characteristics or abilities to use a specific service (such as age); 2. enabling factors, i.e., means whereby a family accesses care (such as social support); and 3. healthcare needs (such as a child’s psychosocial problems). This broad framework is a good fit with the wide-ranging problems experienced by children with CP. Our study is the first to apply this framework to the intensity of care use by these children.

We previously reported that overall care use *was associated with social support and psychosocial problems and that the use of psychosocial care was associated with a child’s age and parenting concerns*, *based on* a cross-sectional study in families with severe complex problems [16]. In the current study with a follow-up design, *we additionally examined changes in intensity of care use in children with and at risk on developing CP*, *ensuring a wide range of intensity of care use*. *Accordingly*, the aim of this study is to identify the changes in the predisposing, enabling and need factors that are associated 1. with a higher likelihood of changes in use of care services and 2. with changes in the intensity of use. The care services use studied comprised *a broad spectrum of general care services including health and psychosocial care*, and also the subset psychosocial care, *including child mental healthcare and child and family services*. *We selected several predisposing characteristics of the child (such as age and gender*, *and also including predetermined factors such as parental education level and adverse life events)*, *as well as enabling (social support and parental care use) and need factors (chronical condition*, *psychosocial problems*, *satisfaction with the parent-child relationship*, *and parenting concerns)*. *This selection was based on the literature regarding determinants impacting the intensity of use of psychosocial care by children*, *as well as on our former study* [4–6; 11–16].

## Method

### Sample and procedure

For this longitudinal study, we followed a cohort of children with CP and their parents, living in an urban setting in the Netherlands. The study was conducted according to the Helsinki regulation. The Medical Ethics Committee at Leiden University decided that approval was not required under Dutch Law (C12.041).

*We aimed to include* parents of children with CP or at risk of developing them with a wide range in intensity of care use living in the community. We recruited these *parents in the general population, using inclusion criteria that concurred with the framework for identifying families with CP [24,25]*. *We included parents when they met the following inclusion criteria*: 1. they had a child between 18 months and 12 years and 2. they experienced at least one of the following conditions: A. the child’s elevated total score on the parent-reported Strengths and Difficulties Questionnaire (SDQ) [26] or Brief Infant Toddler Social Emotional Assessment (BITSEA) [27]; B. persistent parenting concerns as judged by the preventive health care worker and/or parents; C. one or more major life event(s) during the past year as assessed using the *standard screening questionnaire of the well child clinic [28]* and D. care utilization of the child or parent in the past six months. Almost all respondents had three or more of these conditions (97%).

We identified *the respondents* during well-child visits, which are provided in the Netherlands by the preventive youth healthcare services. Attendance rates at these visits are high: 95% of all children [29]. To ensure the inclusion of children who used care with a high intensity, we additionally included children enrolled in specialist child and family services *i*.*e*. *services that are only accessible after referral from primary care*. *Together this study group is expected to represent the whole group of children with CP or at risk of developing them*.

*We used the following inclusion procedure. First, a nurse, doctor or social worker identified parents based on our inclusion criteria, which were embedded in their routine intake questionnaire. Professional care givers then provided oral and written information about the study to the identified families and asked permission from parents to be called by a research assistant. Thereafter, the research assistant asked for informed consent regarding participation in this study*.

Data were collected *by trained research assistants* at two time points, the first in 2013 (T1) and the second 12 months later (T2). Data were collected in a digital questionnaire, although parents could also opt to be interviewed by telephone in the language of their preference. *Parents were reminded three times to fill in this questionnaire and received a gift certificate of 20 euros after doing so*. *Parents were informed that they could withdraw at any moment*.

A total of 512 parents were approached, 354 of whom participated at T1 (response = 69.1%). Of these, 272 participated in the follow-up at 1 year (T1-T2 response = 76.8%), 239 from the well-child clinic group (T1 = 309), and 33 from the group of children using care in a high intensity (T1 = 45). Parents who dropped out at T2 had significantly more sons; more of them were of non-western origin and, on the basis of their home neighborhoods, and more of them had a lower socio-economic position [30].

### Measures

*We used validated questionnaires if available and assessed their reliability in the sample under study*. The children’s service use *and intensity of this use* in the past six months were measured with the Q*uestionnaire Intensive Care for Youth, a questionnaire measuring use of a pre-set list of types of Dutch services [31, 32]. This list has been adapted to the setting of care for youth from the valid and reliable Questionnaire* for Costs Associated with Psychiatric Illnesses and Care Use *(TiC-P) [33,34]. As allowed for by this standard questionnaire, we have added and omitted specific items of care services depending on their relevance for our target population.* Moreover, *respondents had the opportunity to add services we had not listed*. *Services are defined as any care provider or group of care providers*. Dichotomized use at baseline and at follow-up led to four categories expressing change in use, i.e. “never used care”, “stopped using care”, “started using care” and “continued using care”. Intensity of care service use was measured as the number of contacts, defined as planned or unplanned contacts with a professional caregiver by telephone, email, or appointment or home visit; this did not include contacts to make an appointment. We made a distinction between 1. use of any services, which included the use of care delivered in the psychosocial or medical domain; and 2. use of psychosocial services, which included a subset of any care delivered by mental healthcare services, social care services, school care services or family services.

On the basis of Andersen and Newman’s behavioral-health model of access to care, we measured potential determinants of care use, i.e. predisposing, enabling and need factors [21]. We used six predisposing factors: child’s age; parents’ educational level; household composition; child’s ethnicity; parental mental health, and impact of any adverse life events the family had experienced (ALE). Parental mental health status was measured using *the validated* 12-item version of the General Health Questionnaire (Cronbach’s α = .86) [35]. To measure the burden of adverse life events in the previous 12 months, we used the life-events scale of the Brief Instrument Psychological and Pedagogical Problem Inventory (Cronbach’s α = .72) [36].

We measured three enabling factors: partner’s provision of social support, family provision of social support, and care use by a parent. To measure social support, we used two subscales of the *validated Dutch* Family Functioning Questionnaire [37]: “relationship with partner”(Cronbach’s α = .88), and “social functioning of the family” (Cronbach’s α = .91). Parental care use was measured using the TiC-P, similar to the way the child’s care use was measured (see above) [33].

We included four need factors in this study, i.e. a child’s chronic condition; a child’s emotional and behavioral problems; parenting concerns and a parent’s assessment of the quality of their relationship with their child. Questions measuring a child’s chronic health were the following. “Does your child have one or more chronic health condition—such as asthma, diabetes, ADHD or autism—for which treatment is or was needed? What is the impact of this condition on your child’s daily life?” *[38]*. We measured child behavioral and emotional problems using the BITSEA (for children aged between 18 months and 3 years) and the SDQ (for children aged between 3 and 12 years). We constructed the variable as a dichotomy, to be able combine the scores on the different instruments for the whole group. The Dutch versions of both were found to be reliable [26, 39–42]. Cronbach’s α’s *as measured in this study* of the SDQ subscales range from .39 to .74 and Cronbach’s α’s of the two BITSAE subscales on our data were .67 and .80. To measure parenting concerns we used the following question: “In the last 12 months, have you had concerns about your parenting?”[43] Finally, parents’ assessment of their relationship with their child was measured using the subscale of the *validated Dutch* Parenting Load Questionnaire (Cronbach’s α = .83) [44]. *Answers were given in a five point Likert-scale*. *We dichotomized scale sum scores using their medians as cut off*.

### Analyses

First, we described the background characteristics; the scores of the predisposing, enabling and need factors; the use and intensity of any care; and the use and intensity of psychosocial care, all at baseline (T1). Next, on the basis of patterns of use and intensity, we described the changes between baseline and follow-up of predisposing, enabling and need factors for use of any care and use of psychosocial care. Next, we used negative binomial Hurdle modeling to assess the associations between the changes in factors and the changes in care use and its intensity. The score of the dependent variable at T1 was entered as covariate in the Hurdle analyses to be able to address ‘change’ in the outcomes.

Our use of Hurdle modeling was intended to overcome the statistical challenges inherent to data on care use, which typically follow a distribution with many zeroes (no use of care) [45, 46]. Hurdle models have the advantage of estimating two separate parameters in one model to accommodate many zero counts: one dichotomous outcome regarding using care services or not (>0 contacts versus no contacts), and one continuous outcome regarding the number of contacts within the group using care services (>0 contacts). First we assessed the univariate associations of the changes in the independent variables with any care and psychosocial care. On the basis of backward elimination in Hurdle models of all independent variables that were univariately significantly related at the p≤0.1 level, we then assessed which predisposing, enabling and need factors, and changes in these, were associated with changes in use and its intensity. The criterion for removing a factor out from the final models was set at p≤ 0.05. Analyses were performed using SPSS 22.0 [47], and the Hurdle analyses were performed in R, version 3.3.2 [48].

## Results

### Response and respondents’ background characteristics

The sample included more boys than girls, more school-aged children than pre-schoolers, more children of two-parent families than one-parent families, and more children of Dutch ethnicity than of non-Dutch ethnicity (see Table 1). About half of the parents with a high educational level experienced mental health problems and/or *experienced* burden of adverse life events in the previous year.

**Table 1:** Respondents’ baseline characteristics, and care use and intensity of use of any care and of psychosocial care.

	Total	Any care services		Psychosocial care services	
	N^##^	any use	intensity when using mean (SD)^b^	any use	intensity when using mean (SD)^b^
		n (%)^a^		n (%)^a^	
Total	272	203 (75)	21 (35)	121 (45)	25 (39)
Predisposing factors					
Child’s gender					
Boy	152	117 (77)	24 (41)	67 (55)	29 (46)
Girl	120	86 (72)	17 (25)	54 (45)	20 (29)
Child’s age					
Pre-school	107	67 (63)	25 (41)	38 (36)	28 (44)
School-aged	165	111 (67)	15 (23)	87 (53)	15 (19)
Parental educational level					
High	132	93 (71)	24 (39)	53 (40)	18 (26)
Low/ medium	138	109 (79)	16 (26)	67(49)	28 (43)
Household composition					
2-parent family	133	109 (82)	19 (34)	55 (41)	27 (39)
1-parent family	112	73 (65)	22 (37)	49 (44)	24 (45)
Other	23	19 (83)	26 (30)	15 (65)	20 (18)
Ethnicity					
Dutch	155	122 (79)	22 (38)	78 (50)	25 (48)
Western	24	16 (67)	17 (24)	11 (46)	17 (25)
Non-Western	91	64 (70)	20 (33)	31 (34)	28 (38)
Parent had mental health problems					
Yes	150	91 (75)	23 (42)	67 (45)	30 (47)
No	122	112 (75)	18 (25)	54 (44)	19 (26)
Burden of adverse life events					
High	133	100 (76)	26 (40)	64 (48)	30 (44)
Low	124	94 (75)	16 (31)	51 (41)	19 (35)
Enabling factors					
Partner’s provision of social support					
High	140	105 (75)	25 (40)	53 (38)	22 (36)
Low	130	97 (75)	17 (31)	67 (52)	28 (42)
Family provision of social support					
High	139	110 (79)	19 (31)	70 (50)	18 (31)
Low	130	90 (69)	24 (40)	51 (39)	35 (47)
Care use by parent					
Yes	110	88 (80)	26 (38)	54 (49)	30 (40)
No	162	115 (71)	17 (38)	67 (41)	21 (39)
Need factors					
Burden due to chronic condition					
Yes	53	44 (83)	41 (53)	32 (60)	44 (55)
No condition/ no burden	217	159 (73)	25 (26)	89 (41)	18 (29)
Psychosocial problems					
Yes	124	100 (81)	26 (42)	64 (52)	32 (46)
No	138	95 (69)	14 (21)	53 (38)	13 (16)
Parenting concerns					
High	120	90 (75)	30 (44)	60 (50)	35 (47)
Low	152	113 (74)	14 (24)	61 (40)	15 (28)
Parental satisfaction with parent-child relationship					
High	144	115 (80)	33 (19)	61 (42)	22 (36)
Low	127	88 (69)	23 (39)	60 (47)	28 (43)

^##^ N is taken at T1 and n varies due to missing data.

^a^ Respondents using care and the within group percentage.

^b^ Mean and standard deviation of care contacts when a respondent was using care.

#### Care service use and its intensity, and scores on predisposing, enabling and need factors

At baseline, three-quarters of the children in our sample were using some sort of care, and 45% were using psychosocial care (Table 1). For any child using care services, the average intensity of care use was 21 contacts. The intensity of service use was higher if a child was using psychosocial care, with an average of 25 contacts in the previous six months. Predisposing and enabling factors followed a different pattern for ‘any’ and ‘psychosocial care’ use (yes/no) and their intensity (>0 contacts). However, need factors showed the same pattern both for any care use and for psychosocial care use: children whose parents reported a higher score on a need factor used care more often and with a higher intensity than those who reported a lower score.

#### Change in a child’s predisposing, enabling and need factors for care service use and its intensity

Table 2 shows changes in predisposing, enabling and need factors and in care use (yes/no) and its intensity (>0 contacts) over time during the use of care services. Regardless of their difference score on the independent variable, most children were in the “continued care use” category for any care services, and in the “never used care” category for psychosocial care services. More children with an increase in the level of need factors tended to be in the “started care” category than those whose needs were decreased or remained unchanged.

**Table 2:** Descriptives for the change in predisposing, enabling and need factors and any and psychosocial care use and its intensity by children with or at risk of developing CP.

		Care service use (yes/no)				Intensity/number of contacts when using care			
	N^#^	never used care	stopped using care	started using care	continued using care	stopped using care	started using care	continued using care	
								T1	T2
		n (%)^a^	n (%)^a^	n (%)^a^	n (%)^a^	mean (sd)^b^	mean (sd)^b^	mean (sd)^b^	mean (sd) ^b^
***Results for any care***									
Predisposing factors									
Δ Burden of adverse life events									
No change	181	21 (12)	38 (21)	16 (9)	106 (59)	7 (8)	19 (32)	25 (44)	19 (35)
Decrease	57	10 (18)	7 (12)	10 (18)	30 (53)	38 (15)	9 (9)	26 (30)	27 (35)
Increase	9	2 (22)	2 (22)	0	5 (56)	8 (10)	-	8 (11)	16 (13)
Need factors									
Δ Child’s psychosocial problems									
No change	198	29 (15)	39 (20)	22 (11)	108 (55)	9 (12)	15 (28)	22 (34)	21 (30)
Decrease	39	9 (23)	7 (18)	3 (8)	20 (51)	48 (59)	4 (5)	29 (34)	10 (10)
Increase	21	1 (5)	2 (10)	3 (15)	15 (70)	10 (9)	15 (6)	21 (15)	35 (64)
ΔParenting concerns									
No change	197	26 (13)	37 (18)	17(9)	117 (59)	10 (13)	17 (32)	26 (41)	19 (24)
						(911)			
Decrease	17	3 (18)	3 (18)	2 (12)	9 (53)	5(3)	10 (11)	20 (22)	58 (92)
Increase	57	11 (19)	13 (23)	10 (18)	23 (40)	14 (24)	8 (8)	20 (36)	16 (30)
***Results for psychosocial care***									
Predisposing factors									
Child’s age^d^									
Pre-school	107	54 (50)	15 (14)	21 (20)	17 (16)	14 (22)	8 (13)	17 (16)	19 (18)
School-aged	165	51 (31)	27 (16)	25 (15)	62 (38)	12 (11)	10 (12)	36 (51)	22 (30)
Δ Burden of adverse life events									
No change	181	70 (39)	31 (17)	31 (17)	49 (27)	8 (8)	10 (14)	38 (54)	23 (32)
Decrease	57	19 (33)	4 (7)	12 (21)	22 (39)	36 (10)	7 (8)	25 (30)	19 (22)
Increase	9	4 (44)	2 (22)	0	3 (27)	7 (9)	-	14 (12)	13 (14)
Need factors									
Δ Child’s psychosocial problems^c^									
No change	198	78 (39)	30 (15)	33 (17)	57 (29)	11 (12)	12 (14)	26 (36)	22 (30)
Decrease	39	16 (41)	6 (15)	7 (18)	10 (26)	8 (8)	3 (3)	44 (56)	13 (11)
Increase	21	7 (33)	3 (15)	4 (19)	7 (33)	30 (41)	8 (4)	15 (18)	28 (24)
Δ Parenting concerns^c^									
No change	197	75 (38)	28 (14)	28 (14)	66 (34)	13(13)	12 (15)	31 (48)	20 (22)
Decrease	17	6 (35)	3(18)	4 (23)	4 (23)	3 (2)	6 (3)	27 (29)	57 (82)
Increase	57	24 (42)	10 (18)	14 (24)	9 (16)	15 (12)	6 (8)	37(46)	13 (19)
									14 (87)

^##^ n varies due to missing data.

^a^ Respondents using care and the within group-percentage.

^b^ Mean and standard deviation of care contacts when a respondent who used care.

Regarding change in the intensity of care service use (>0 contacts), we found in the “continuing care” category that children whose need factors had decreased also showed a decrease in the intensity of care use. In line with this finding, children with an increase in need factors also showed an increase in the intensity of care in the “continuing care” category. However, we did not find the same relationship for parenting concerns.

Table 3 shows the final Hurdle models regarding the multivariate associations of change in independent variables with change in use of any and psychosocial care, and their intensity (see S1 Appendix for the results of the univariate regression models for all factors). First we discuss the zero part of the Hurdle models regarding care use (yes/no). The final model regarding use of any care consisted of burden of adverse life events (ALE) (a predisposing factor), and parenting concerns (a need factor). Whereas a decrease in ALE was associated with lower odds of change of care use, an increase in parenting concerns was associated with higher odds. Regarding psychosocial care use, the final model consisted of the same factors as use of any care, but with the addition of child’s age. School-aged children had higher odds on change of psychosocial care use than did pre-school children.

**Table 3:** Final Hurdle models for change in factors associated with change in care use and its intensity by children with CP using care: Multivariate odds ratios for changes in care use and rate ratios for changes in intensity of care use for any care and for psychosocial care services.

	Δ Care service use (yes/no)	Δ Intensity/number of contacts when using care
	adj. OR (95% CI)^a^^b^	adj. RR (95% CI)^a^^c^
***Final model for any care***^*>>*^		
Predisposing factors		
Δ Burden of adverse life events^d^	0.94 (0.90;0.99)*	0.95 (0.92;0.98)**
Need factors		
Δ Child’s psychosocial problems^d^		
No change	Ref (1)	Ref (1)
Decrease	0.73 (0.32;1.68)	0.38 (0.20;0.73)**
Increase	3.27 (0.69;15.48)	1.17 (0.54;2.56)
Δ Parenting concerns^c^	1.29 (1.11;1.51) ***	1.13 (0.99;1.29)
***Final model for psychosocial care***^*>>*^		
Predisposing factors		
Child’s age^d^		
Pre-school	Ref (1)	Ref (1)
School-aged	1.99 (1.09;3.63)*	1.32 (0.72;2.43)
Δ Burden of adverse life events^d^	0.93 (0.89;0.97)***	0.98 (0.95;1.01)
Need factors		
Δ Child’s psychosocial problems^e^		
No change	Ref (1)	Ref (1)
Decrease	0.84 (0.36;1.97)	0.39 (0.18;0.84)*
Increase	1.02 (0.36;2.92)	1.16 (0.46;2.90)
Δ Parenting concerns^c^	1.26 (1.10;1.45)**	1.08 (0.95;1.24)

^a^ Backward stepwise regression analyses were conducted with the difference score of the factor, if available, and care use at T1 as covariate. The factors entered were parental educational level, child’s age, burden of adverse life events, partner’s provision of social support, child’s chronic condition, child’s psychosocial problems, and parenting concerns. The criterion for removing a factor from the model was set at P-value>0.05

^b^ Predictors were removed in the following order: chronic condition, parental educational level, and partner’s provision of social support

^c^ Only one factor, chronic condition, was removed from the model.

^d^ These factors are constructed as difference-of-scale scores between T2-T1.

^e^ This factor is constructed as difference of dichotomized scores between T2-T1.

*p<0.05

**p<0.01

***p<0.001.

Next, we discuss the count part of the Hurdle analyses (>0 contacts). Regarding the intensity of any care use, the final model consisted of burden of adverse life events (a predisposing factor), and psychosocial problems (a need factor). A child’s decrease in ALE was associated with decreased intensity of use, and a child’s decrease in psychosocial problems was associated with decreased intensity of psychosocial care, in comparison with children with no changes in their level of problems. The final model for psychosocial care services consisted only of psychosocial problems (a need factor), with associations similar to those for any care.

## Discussion

This study shows that changes in the predisposing and need factors of Andersen and Newman’s behavioral-health model of access to care were relevant to explaining changes in care use and its intensity by children with CP or at risk of developing it. However, enabling factors were not. We also found that care use was related to factors other than changes in its intensity. Relative to the situation at baseline, when children experienced a diminished burden of life events (ALE) or when more parenting concerns were reported at follow-up, children were less likely to use any care or psychosocial care. School-aged children were also more likely than pre-schoolers to use psychosocial care. The intensity of any care use and of psychosocial care use decreased when the degree of psychosocial problems decreased. The intensity of any care use also decreased when ALE decreased. Moreover, where ALE was associated both with care use and with its intensity, parenting problems uniquely impacted care use and psychosocial problems uniquely impacted its intensity.

We found that several changes in predisposing *(i*.*e*. *burden of ALE and a child’s age)* and need factors *(i*.*e*. *parenting concerns and psychosocial problems)* were associated with changes in care use and its intensity, both for overall care use and for the use of psychosocial services, *but that changes in enabling factors were not*. The determinants we found are in line with previous findings *[49–55].* An explanation may be that enabling factors are harder to change than predisposing and need factors *in the relative short time span of our study (one year)*. *For example*, *it is more difficult for a child social worker to convince parents to make use of mental health care for their own mental problems than to address parenting concerns*. This study shows the value of the Andersen and Newman model for studying the intensity of care use, especially in distinguishing enabling factors from other factors affecting families with a child at risk of CP or of developing them.

The results of this study added burden of ALE as a factor impacting change in intensity of care use. *Research showed that ALE is an important determinant of care use in general [56, 57]. ALE will especially affect children with CP, interacting strongly with the other problems of these children, thereby leading to more intense problems.* Unexpectedly, we found a slight negative relative risk between ALE and the intensity of any care use. *We noted that the burden of ALE decreased in a relatively large group of children while they were using care*. *Children with CP may have been motivated to continue treatment even when the burden of ALE decreased, because trauma-based therapies are known to have a positive effect on other emotional conditions [58,59]. Furthermore, when the safety of a child is at risk, as in cases of domestic violence, care professionals will ideally continue treatment to monitor the situation.* Our results indicate that change in ALE is relevant to the whole care process, i.e. not only care use itself, but also to its intensity.

*Although improving social support is at the core of treatment of families with complex problems, in the final models of our study this factor was absent [60]. It can be hypothesized that social support works differently for families with complex problems than for the general population [16, 61–63]. The families’ social networks in case of CP are usually large and suitable for dealing with daily challenges of living with a child with complex problem[61–63]. However, regardless of their perceived social support, families will turn to professionals to bring about long-term improvements, surmising that they may not be able to achieve these improvements with their own network. Also, professionals may not yet have managed to bring about changes in the quality of support by the social environment because of the relatively short period of our study (one year).* Although social support is a known determinant impacting a child’s care use, more research is needed to understand how to optimize its impact for families of a child with CP.

Finally, we found that changes that changes in intensity with which care is used (>0 contacts) were affected by factors other than changes in care use in itself (yes/no). This supports earlier findings in the scarce research available on intensity of care use [8–10]. For both any care and psychosocial care, our study shows that parents with parenting concerns were more likely to use care, and the intensity of care use increased when there were psychosocial problems. Both need factors are known drivers of help-seeking behavior [64]. Our study showed that parenting concerns impacted care use but not intensity, while a child’s psychosocial problems were relevant to intensity rather than to care use.

### Strengths and limitations

A major strength of this study is its comprehensive use of the data, obtained by using the Hurdle model. This model overcomes the difficulties inherent to using a single model to assess factors that impact care use and its intensity, which cannot be assessed by mainstream generalized linear models. We therefore believe that the use of Hurdle models provides added value for researchers interested in care utilization. *Another strength of the study is that the study group of children with or at risk of developing CP were living in the community*, *including children in treatment with different intensities of care use or not using care at all*. *In most other research the study groups are limited to children with CP who are using a specific treatment [8,25].*

A limitation of this study concerned some small selective loss to follow-up. A relatively high number of children who were lost to follow-up were boys and had parents of non-western origin. *Another limitation is that we used a self-report questionnaire to establish care use in the previous six months*. *This may have caused some recall bias*, *especially for intensity of care use and the determinants burden of ALE and impact of chronical conditions of the child*. *This may have added measurement error and thus a weakening of reported associations*, *probably without clear under- or overestimation*.

### Implications for practice

A new finding in this study is the effect that the burden of ALE has on the intensity of care use, a factor that is relevant to the whole care-seeking process, i.e. not only entering care, but also the intensity of its use. This shows the importance of providing interventions that focus on the effects of ALE, on the impact of these effects on intensity, and thus on the costs of care [58, 59]. For this reason, those who assess and treat children with CP should pay close attention to adverse life events and the way children and their families deal with them.

We also found that, while a decline in psychosocial problems was associated with a decrease in intensity of care use, care use in itself was not affected by changes in psychosocial problems. Conceivably, various barriers hinder the process of starting care. In their recent systematic review, in which they provide an overview of the barriers facing children with or at risk of developing CP, Reardon and colleagues show how insufficient knowledge and understanding of psychosocial problems and the help-seeking process on the part of parents is a core component that hinders care use [65]. Policymakers and professional care providers should make efforts to educate parents on recognizing their child’s psychosocial problems, and also on the local pathways to help.

### Implications for further research

With regard to care use and its intensity in this group of children, our study shows the enabling factors defined by Andersen and Newman to be less relevant than the predisposing and need factors [8]. To understand the contribution and any possible indirect impact of enabling factors, further research is required. We therefore have two recommendations: 1. a larger respondent group (to accommodate mediation analysis); and 2. extension of the time-lapse in the longitudinal design.

*Regarding the enabling factor social support, more research is needed on how to improve the quality of support provided by the network of families with a child with complex problems. We advise the development and evaluation of a treatment module for parents and key persons in their social network to improve support skills. These new skills can be thought by volunteers who are able to model healthy support*.

In this group of children we also found that the intensity of use of care services is affected by factors different from those influencing the use of care in itself. Understanding the mechanism underlying the intensity of care use can help the *development of more effective and efficient pathways to care for children with or at risk of developing CP*. This will require further research into this mechanism behind care use and its intensity by children with CP.

### Conclusion

With regard to the use of any care, or psychosocial care, and the intensity of this care by children who with or at risk of developing CP, our study shows that changes in predisposing factors (i.e., a child’s age and burden of life events) and need factors (i.e., a child’s psychosocial problems and parenting concerns) are associated with change in use or intensity of use, and enabling factors are not. The importance of effective treatment of ALE is emphasized by the fact that ALE are a factor that contributes to the intensity of care use. The level of a child’s psychosocial problems is also relevant to the intensity of care use (>0 contacts), but not to the use of care in itself (yes/no). To improve care use by children with these needs, policymakers should address parents’ knowledge with regard to identifying psychosocial problems and the help-seeking process. Finally, our findings demonstrate the added value of studying the intensity of care use, especially on the basis of Andersen and Newman’s model of care-seeking. Such study will improve our insight into the drivers of the intensity of care use by children with CP.

## Supporting information

S1 Appendix Results of the hurdle analyses. (DOCX)

S2 Appendix Interview protocol for the project oké in Den Haag. (DOCX)

S1 File (PDF)

S2 File (DOCX)

## References

[1] Denholm R, Power C, Li L, Thomas C. Child maltreatment and household dysfunction in a British Birth Cohort. Child Abuse Rev 2013;22(5):340–353.

[2] Stith SM, Liu T, Davies LC, Boykin EL, Alder MC, Harris JM, et al (2009). Risk factors in child maltreatment: A meta-analytic review of the literature. Aggression and Violent Behavior, 14(1), 13–29. 10.1016/j.avb.2006.03.006

[3] Lucas PJ, McIntosh K, Petticrew M, Roberts HM, Shiell A. Financial benefits for child health and well-being in low income or socially disadvantaged families in developed world countries. Cochrane Database of Systematic Reviews 2008(2). 10.1002/14651858.CD006358.pub2 PMC8923519 18425950

[4] Mendenhall E, Kohrt BA, Norris SA, Ndetei D, Prabhakaran D. Non-communicable disease syndemics: poverty, depression, and diabetes among low-income populations. The Lancet 2017 4–10 3 2017; 389(10072):951–963. 10.1016/S0140-6736(17)30402-6 PMC5491333 28271846

[5] Gawande A. The Hot Spotters, can we lower medical costs by giving the neediest patients better care? The New Yorker, Januari 17^th^ 2011: 40–51. 21717802

[6] Hayden C, Jenkins C. Children taken into care and custody and the 'troubled families' agenda in England. Child Fam Soc Work 2015;20(4):459–469.

[7] Hayden C, Jenkins C. ‘Troubled Families’ Programme in England: ‘wicked problems’ and policy-based evidence. Policy Stud 2014;35(6):631–649.

[8] Tausendfreund T, Knot-Dickscheit J, Post WJ, Knorth EJ, Grietens H. Outcomes of a coaching program for families with multiple problems in the Netherlands: A prospective study. Children and Youth Services Review 2014 11;46:203–212.

[9] Goerge RM, Smithgall C, Seshadri R, Ballard P. llinois Families and Their Use of Multiple Service Systems. Chicago: Chapin Hall at the University of Chicago; 2010.

[10] Sacco FC, Twemlow SW, Fonagy P. Secure attachment to family and community: a proposal for cost containment within high user populations of multiple problem families Smith College Studies in Social Work 2008.

[11] Nanninga M, Jansen DEMC, Knorth EJ, Reijneveld SA. Enrolment of children and adolescents in psychosocial care: more likely with low family social support and poor parenting skills. Eur Child Adolesc Psychiatry 2015;24 (4):407–416. 10.1007/s00787-014-0590-3 25116036

[12] Burns BJ, Phillips SD, Wagner HR, Barth RP, Kolko DJ, Campbell Y, et al Mental health need and access to mental health services by youths involved with child welfare: A national survey. J Am Acad Child Adolesc Psychiatry 2004;43(8):960–970. 10.1097/01.chi.0000127590.95585.65 15266190

[13] Verhulst FC, Van Der Ende J. Factors associated with child mental health service use in the community. J Am Acad Child Adolesc Psychiatry 1997;36(7):901–909. 10.1097/00004583-199707000-00011 9204667

[14] Tick NT, Van Der Ende J, Verhulst FC. Ten-year increase in service use in the Dutch population. European Child and Adolescent Psychiatry 2008;17(6):373–380. 10.1007/s00787-008-0679-7 18427867

[15] Tausendfreund T, Knot-Dickscheit J, Schulze GC, Knorth EJ, Grietens H. Families in multi-problem situations: Backgrounds, characteristics, and care services. Child Youth Serv 2016;37(1):4–22.

[16] Pannebakker NM, Kocken PL, Theunissen MHC, van Mourik K, Crone MR, Numans ME, et al Services use by children and parents in multiproblem families. Child Youth Serv Rev 2018;84:222–228.

[17] Farmer EMZ, Stangl DK, Burns BJ, Costello EJ, Angold A. Use, persistence, and intensity: Patterns of care for children's mental health across one year. Community Ment Health J 1999;35(1):31–46. 10.1023/a:1018743908617 10094508

[18] Hamilton HA, Paglia-Boak A, Wekerle C, Danielson AM, Mann RE. Psychological Distress, Service Utilization, and Prescribed Medications among Youth with and without Histories of Involvement with Child Protective Services. Int J Ment Health Addict 2011;9(4):398–409.

[19] Saurina C, Vall-Llosera L, Saez M. Factors determining access to and use of primary health care services in the Girona Health Region (Spain). Eur J Health Econ 2012;13(4):419–427. 10.1007/s10198-011-0313-3 21499790

[20] Palacio-Vieira J, Villalonga-Olives E, Valderas JM, Herdman M, Alonso J, Rajmil L. Predictors of the use of healthcare services in children and adolescents in Spain. International Journal of Public Health 2013;58(2):207–215 10.1007/s00038-012-0360-2 22552748

[21] Andersen R, Newman JF. Societal and individual determinants of medical care utilization in the United States. Milbank mem fd quart 1973;51(1):95–124. 4198894

[22] Phillips KA, Morrison KR, Andersen R, Aday LA. Understanding the context of healthcare utilization: Assessing environmental and provider-related variables in the behavioral model of utilization. Health Serv Res 1998;33(3 I):571–596 9685123 PMC1070277

[23] Babitsch B, Gohl D, von Lengerke T. Re-revisiting Andersen's Behavioral Model of Health Services Use: a systematic review of studies from 1998–2011. Psychosoc Med. 2012;9. 10.3205/psm000089 PMC3488807 23133505

[24] Bodden DHM, Dekovitch M. Multiprobleemgezinnen ontrafeld [multiproblem families discovered]. Tijdschrift voor Orthopedagogiek 2010;49:259–271.

[25] Bodden DHM, Dekovic M. Multiproblem Families Referred to Youth Mental Health: What's in a Name? Fam Process 2016;55(1):31–47. 10.1111/famp.12144 25754003

[26] Goodman R. The strengths and difficulties questionnaire: A research note. J Child Psychol Psychiatry Allied Disciplines 1997;38(5):581–586. 10.1111/j.1469-7610.1997.tb01545.x 9255702

[27] Briggs-Gowan MJ, Carter AS, Irwin JR, Wachtel K, Cicchetti DV. The Brief Infant-Toddler Social and Emotional Assessment: Screening for Social-Emotional Problems and Delays in Competence. J Pediatr Psychol 2004;29(2):143–155. 10.1093/jpepsy/jsh017 15096535

[28] Theunissen M., Pas van der S. & Harten van L. Achtergronddocument: Een uniform triageprotocol voor het signaleren van ontwikkelings- of gezondheidsrisico’s bij basisschoolkinderen door de Jeugdgezondheidszorg [Background information: a uniform protocol for discovering a triage of risks on developmental and health problems by child preventive health care]. 2019; 060 16673.

[29] CBS Statistics Netherlands. Ouders geven consultatiebureau gemiddeld een ruime 7 [Parents appreciatie well visit clinics]. 2014; Available at: www.cbs.nl/nl-nl/nieuws/2014/44/ouders-geven-consultatiebureau-gemiddeld-een-ruime-7.

[30] Sociaal Cultureel Planbureau. social economic status. 2015 August 18th.

[31] Bouwmans CAM, Schawo SJ, Jansen DEMC, Vermeulen KM, Reijneveld SA, Hakkaart-van Roijen L. Handleiding Vragenlijst Intensieve Jeugdzorg: Zorggebruik en productieverlies [Manual Questionnaire Intensive Care for Youth: health care utilization and productivity loss]. Rotterdam: Erasmus MC; 2012.

[32] Jansen DE, Vermeulen KM, Schuurman-Luinge AH, Knorth EJ, Buskens E, Reijneveld SA. Cost-effectiveness of Multisystemic Therapy for adolescents with antisocial behavior: Study protocol of a randomized controlled trial. BMC Public Health 2013;13(1). 10.1186/1471-2458-13-369 PMC3641956 23597036

[33] Hakkaart-Van Roijen L, Van Straten A, Donker M, Tiemens B. Trimbos/iMTA questionnaire for Costs associated with Psychiatric Illness (TiC-P). Rotterdam; 2002.

[34] Bouwmans C, De Jong K, Timman R, Zijlstra-Vlasveld M, Van Der Feltz-Cornelis C, Tan SS, et al Feasibility, reliability and validity of a questionnaire on healthcare consumption and productivity loss in patients with a psychiatric disorder (TiC-P). BMC Health Serv Res 2013;13(1). 10.1186/1472-6963-13-217 PMC3694473 23768141

[35] Koeter MWJ, Ormel J. General health questionnaire, manual [Nederlandse bewerking, Handleiding]. Lisse: Swets & Zeitlinger; 1991.

[36] De Wolff MS, Theunissen MHC, Vogels AGC, Reijneveld SA. Three questionnaires to detect psychosocial problems in toddlers: A comparison of the BITSEA, ASQ:SE, and KIPPPI. Academic Pediatrics 2013;13(6):587–592. 10.1016/j.acap.2013.07.007 24238686

[37] Van der Ploeg JD, Scholte EM. Handleiding Vragenlijst Gezinsfunctioneren (GVL) [Manual Family Functioning Questionnaire]. Houten: Bohn Stafleu Van Loghum; 2008.

[38] Wingerd M. Gezondheidsenquete-2013 [Dutch Health care questionnaire]. 12-02-2020; Available at: www.cbs.nl/nl-nl/achtergrond/2015/15/gezondheidsvragenlijst-gezondheidsenquete-2015

[39] Kruizinga I, Jansen W, de Haan CL, van der Ende J, Carter AS, Raat H. Reliability and validity of the Dutch version of the brief infant-toddler social and emotional assessment (BITSEA). PLoS ONE 2012;7(6). 10.1371/journal.pone.0038762 PMC3371000 22715411

[40] Theunissen MHC, Vogels AGC, De Wolff MS, Reijneveld SA. Characteristics of the strengths and difficulties questionnaire in preschool children. Pediatrics 2013; 131(2):e446–e454. 10.1542/peds.2012-0089 23296429

[41] Vogels AG, Crone MR, Hoekstra F, Reijneveld SA. Comparing three short questionnaires to detect psychosocial dysfunction among primary school children: A randomized method. BMC Public Health 2009;9. 10.1186/1471-2458-9-489 PMC2804619 20035636

[42] Mieloo C, Raat H, van Oort F, Bevaart F, Vogel I, Donker M., et al (2012). Validity and reliability of the strengths and difficulties questionnaire in 5–6 year olds: Differences by gender or by parental education? PLoS ONE, 7:5. 10.1371/journal.pone.0036805 PMC3356337 22629332

[43] Reijneveld S. A., De Meer G., Wiefferink C. H., & Crone M. R. (2008). Parents' concerns about children are highly prevalent but often not confirmed by child doctors and nurses. *BMC Public Health*, 8 10.1186/1471-2458-8-124 18423036 PMC2383909

[44] Vermulst A, Kroes G, De Meyer RE, Nguyen RE, Veerman JW. Opvoedingsbelastingvragenlijst (OBVL) Handleiding [The Parenting Load Questionnaire, instruction manual]. Nijmegen: Radboud Universiteit Nijmegen / Praktikon; 2012.

[45] Hofstetter H, Dusseldorp E, Zeileis A, Schuller AA. Modeling Caries Experience: Advantages of the use of the hurdle model. Caries Res 2016;50(6):517–526. 10.1159/000448197 27639918

[46] Lee AH, Wang K, Scott JA, Yau KKW, McLachlan GJ. Multi-level zero-inflated poisson regression modelling of correlated count data with excess zeros. Stat Methods Med Res 2006;15(1):47–61. 10.1191/0962280206sm429oa 16477948

[47] IBM corp. IBM SPSS Statistics for Windows. 2013.

[48] R Core Team. R: A language and environment for statistical computing. 2013.

[49] Wu P, Hoven CW, Bird HR, Moore RE, Cohen P, Alegria M, et al Depressive and disruptive disorders and mental health service utilization in children and adolescents. J Am Acad Child Adolesc Psychiatry 1999;38(9):1081–1090. 10.1097/00004583-199909000-00010 10504806

[50] Janicke DM, Finney JW. Determinants of children's primary health care use. Journal of Clinical Psychology in Medical Settings 2000;7(1):29–39.

[51] Riley AW, Finney JW, Mellits ED, Starfield B, Kidwell S, Quaskey S, et al Determinants of children’s health care use: An investigation of psychosocial factors. Med Care 1993;31(9):767–783. 10.1097/00005650-199309000-00002 8366679

[52] Stahmer AC, Leslie LK, Hurlburt M, Barth RP, Webb MB, Landsverk J, et al Developmental and behavioral needs and service use for young children in child welfare. Pediatrics 2005;116(4):891–900. 10.1542/peds.2004-2135 16199698 PMC1550707

[53] Welkom JS, Hilliard ME, Rand CS, Eakin MN, Riekert KA. Caregiver depression and perceptions of primary care predict clinic attendance in head start children with asthma. Journal of Asthma 2015;52(2):176–182. 10.3109/02770903.2014.956891 25144553 PMC5858552

[54] Fernandez E. Supporting children and responding to their families: Capturing the evidence on family support. Child Youth Serv Rev 2007;29(10):1368–1394.

[55] Martinez JI, Lau AS. Do Social Networks Push Families Toward or Away From Youth Mental Health Services?: A National Study of Families in Child Welfare. J Emot Behav Disord 2011;19(3):169–181. 10.1177/1063426610377898 27076777 PMC4828040

[56] Norman RE, Byambaa M, De R, Butchart A, Scott J, Vos T. The Long-Term Health Consequences of Child Physical Abuse, Emotional Abuse, and Neglect: A Systematic Review and Meta-Analysis. PLoS Med 2012;9(11). 10.1371/journal.pmed.1001349 PMC3507962 23209385

[57] Hughes K, Bellis MA, Hardcastle KA, Sethi D, Butchart A, Mikton C, et al The effect of multiple adverse childhood experiences on health: a systematic review and meta-analysis. Lancet Public Health 2017;2(8):e356–e366. 10.1016/S2468-2667(17)30118-4 29253477

[58] Schneider SJ, Grilli SF, Schneider JR. Evidence-based treatments for traumatized children and adolescents. Curr Psychiatry Rep 2013;15(1).-012-0332-5 10.1007/s11920-012-0332-5 23250813

[59] Connor DF, Ford JD, Arnsten AFT, Greene CA. An update on posttraumatic stress disorder in children and adolescents. Clin Pediatr 2015;54(6):517–528. 10.1177/0009922814540793 24990362

[60] Stanhope V, Videka L, Thorning H, McKay M. Moving Toward Integrated Health: An Opportunity for Social Work. Soc Work Health Care 2015;54(5):383–407. 10.1080/00981389.2015.1025122 25985284

[61] Matos AR, Sousa LM. How multiproblem families try to find support in social services. J Soc Work Pract 2004;18(1):65–80.

[62] Sousa L. Building on personal networks when intervening with multi-problem poor families. J Soc Work Pract 2005;19(2):163–179.

[63] Sousa L, Rodrigues S. Linking formal and informal support in multiproblem low-income families: The role of the family manager. J Community Psychol 2009;37(5):649–662.

[64] Pohlmeier W, Ulrich V. An econometric model of the two-part decision making process in the demand for healthcare. J Hum Resour 1995;30(2):339–361.

[65] Reardon T, Harvey K, Baranowska M, O’Brien D, Smith L, Creswell C. What do parents perceive are the barriers and facilitators to accessing psychological treatment for mental health problems in children and adolescents? A systematic review of qualitative and quantitative studies. Eur Child Adolesc Psychiatry 2017;26(6):623–647. 10.1007/s00787-016-0930-6 28054223 PMC5446558

