# Supplementary material for: Care use and its intensity in children with complex problems are related to varying child and family factors: A follow-up study
Source: PLoS One. 2020 May 6;15(5):e0231620. doi: 10.1371/journal.pone.0231620 (PMC7202640; doi:10.1371/journal.pone.0231620)
Supplement: S1 Appendix — (DOCX) [file pone.0231620.s001.docx]

**Appendix Results of the Hurdle analyses**

Table A1: Hurdle regression analyses for change in factors associated with change in care use and its intensity analyses:

univariate and multivariate odds ratios for change in care use and rate ratios for the change in intensity of care use for any care use by children with CP

|  | $\Delta$**care services use**  **unadj. OR (95% CI)^a^** | $\Delta$**intensity of use**  **unadj. RR (95% CI)^a^** | $\Delta$**care services use**  **adj. OR (95% CI)^bc^** | $\Delta$**intensity of use**  **adj. RR (95% CI) ^bc^** |
| --- | --- | --- | --- | --- |
| **Predisposing factors** |  |  |  |  |
| Child’s gender ^e^ |  |  |  |  |
| Boy vs. girl | 0.97 (0.58; 1.59) | 0.82 (0.49; 1.35) |  |  |
| Child’s age ^e^ |  |  |  |  |
| Pre-school v. school-aged | 1.68 (0.74; 2.04) | 1.16 (0.69; 1.93) |  |  |
| Parental educational level ^e^ |  |  |  |  |
| High vs. low/ medium | 1.06 (0.65; 1.76) | .63 (0.38; 1.04)^ |  |  |
| Household composition ^e^ |  |  |  |  |
| 2-parent family | Ref (1) | Ref (1) |  |  |
| 1-parent family | 1.06 (0.63; 1.81) | 0.83 (0.49; 1.40) |  |  |
| Other | 0.85 (0.34; 2.11) | 1.86 (0.71; 4.92) |  |  |
| Ethnicity ^e^ |  |  |  |  |
| Dutch | Ref (1) | Ref (1) |  |  |
| Western | 0.95 (0.38; 2.37) | 1.58 (0.65;3.83) |  |  |
| Non-western | 0.76 (0.44;1.31) | 1.27 (0.73;2.18) |  |  |
| $\Delta$Parent had a mental-health problem ^d^ | 1.07 (0.98;1.16) | 0.98 (0.90;1.06) |  |  |
| $\Delta$Impact of life events ^d^ | 0.97 (0.93; 1.00)^ | 0.97 (0.95; 1.00)^ | 0.94 (0.90;0.99)* | 0.95 (0.92;0.98)** |
| **Enabling factors** |  |  |  |  |
| $\Delta$Partner’s provision of social support ^d^ | 0.94 (0.88; 1.00)^ | 1.05 (1.00;1.11)^ |  |  |
| $\Delta$Family’s provision of social support ^d^ | 0.98 (0.94;1.03) | 1.00 (0.97;1.04) |  |  |
| $\Delta$Care provided to parent ^e^ |  |  |  |  |
| No difference | Ref (1) | Ref (1) |  |  |
| Increase | 1.28 (0.68;1.72) | 0.87 (0.46;1.62) |  |  |
| Decrease | 0.66 (0.43; 1.78) | 0.81 (0.38;1.73) |  |  |
| **Need factors** |  |  |  |  |
| $\Delta$Child’s chronic condition ^e^ |  |  |  |  |
| No difference | Ref (1) | Ref (1) |  |  |
| Increase | 3.2 (0.67;4.15) | 0.62 (0.31;1.25) |  |  |
| Decrease | 1.67 (1.18;8.68)* | 1.21 (0.53;5.75) |  |  |
| $\Delta$Child’s psychosocial problems ^e^ |  |  |  |  |
| No difference | Ref (1) | Ref (1) | Ref (1) | Ref (1) |
| Increase | 3.14 (0.89;11.0)^ | 1.74 (0.76;3.98) | 3.27 (0.69;15.48) | 1.17 (0.54;2.56) |
| Decrease | 0.75 (0.37;1.52) | 0.40 (0.20;.83)* | 0.73 (0.32;1.68) | 0.38 (0.20;0.73)** |
| $\Delta$Parenting concerns ^d^ | 1.24 (1.10;1.41)*** | 1.08 (0.98;1.18) | 1.29 (1.11;1.51) *** | 1.13 (0.99;1.29) ^ |
| $\Delta$Parental satisfaction relationship child ^d^ | 0.93 (0.83; 1.04) | 1.00 (0.90; 1.12) |  |  |

^a^ Univariate regression analyses with factors at T1 and as covariate care use or the intensity of care use, i.e. number of contacts at T2; ^b^ Backward stepwise regression analyses were conducted with the difference score of the factor, if available, and care use at T1 as covariate. The criteria for taking a factor out of the model was set at P-value > 0.05. ^c^ Predictors were taken out in following order: chronical condition, parental educational level and partner’s provision of social support. ^d^ These factors are constructed as difference-of-scale scores between T2-T1.^e^ This factor is constructed as difference of dichotomic scores between T2-T1. *p<0.05 **p<0.01 ***p<0.001.

Table A2: Hurdle regression analyses for change in factors associated with change in care use and its intensity analyses:

univariate and multivariate odds ratios for change in care use and rate ratios for the change in intensity of care use for psychosocial care use by children with CP

|  | | $\Delta$**care services use**  **unadj. OR (95% CI)^a^** | $\Delta$**intensity of use**  **unadj. RR (95% CI)^a^** | $\Delta$**care services use**  **adj. OR (95% CI)^bc^** | $\Delta$**intensity of use**  **adj. RR (95% CI) ^bc^** |
| --- | --- | --- | --- | --- | --- |
| **Predisposing factors** |  |  |  |  |  |
| Child’s gender ^e^ | |  |  |  |  |
| Boy vs. girl | | 1.21 (0.73;2.02) | 0.83 (.19;1.42) |  |  |
| Child’s age ^e^ | |  |  |  |  |
| Pre-school v. school-aged | | 1.64 (0.98;2.77)^ | 1.19 (0.66;2.14) | 1.99 (1.09;3.63)* | 1.32 (0.72;2.43) |
| Parental educational level ^e^ | |  |  |  |  |
| High vs. low/ medium | | 0.73 (0.44;1.21) | 0.83 (0.49;1.41) |  |  |
| Household composition ^e^ | |  |  |  |  |
| 2-parent family | | Ref (1) | Ref (1) |  |  |
| 1-parent family | | 1.52 (0.89;2.60) | 0.87 (0.50 ;1.49) |  |  |
| Other | | 2.12 (0.82;5.49) | 1.81 (0.77;4.26) |  |  |
| Ethnicity ^e^ | |  |  |  |  |
| Dutch | | Ref (1) | Ref (1) |  |  |
| Western | | 0.83 (0.33;2.06) | 0.76 (0.28;2.05) |  |  |
| Non-western | | 0.85 (0.49;1.47) | 0.97 (0.54;1.74) |  |  |
| $\Delta$Parent had a mental-health problem ^d^ | | 1.04 (0.96;1.14) | 0.94 (0.85;1.05) |  |  |
| $\Delta$Impact of life events ^d^ | | 0.95 (0.91;0.98)** | 1.00 (0.97;1.03) | 0.93 (0.89;0.97)*** | 0.98 (0.95;1.01) |
| **Enabling factors** | |  |  |  |  |
| $\Delta$Partner’s provision of social support ^d^ | | 0.97 (0.91;1.03) | 1.04 (0.99;1.10) |  |  |
| $\Delta$Family’s provision of social support ^d^ | | 1.00 (0.96;1.04) | 0.99 (0.96;1.03) |  |  |
| $\Delta$Care provided to parent ^e^ | |  |  |  |  |
| No difference | | Ref (1) | Ref (1) |  |  |
| Increase | | 1.03(0.54;1.96) | 1.11 (0.56;2.20) |  |  |
| Decrease | | 0.65 (0.31;1.36) | 0.61 (0.27;1.38) |  |  |
| **Need factors** | |  |  |  |  |
| $\Delta$Child’s chronic condition ^e^ | |  |  |  |  |
| No difference | | Ref (1) | Ref (1) |  |  |
| Increase | | 2.45 (1.11;5.38)* | 1.31 (0.41;1.88) |  |  |
| Decrease | | 1.51 (0.64;3.57) | 0.87 (0.55;3.08) |  |  |
| $\Delta$Child’s psychosocial problems ^e^ | |  |  |  |  |
| No difference | | Ref (1) | Ref (1) | Ref (1) | Ref (1) |
| Increase | | 1.30 (0.51;3.34) | 1.28 (0.52;3.18) | 1.02 (0.36;2.92) | 1.16 (0.46;2.90) |
| Decrease | | 0.87 (0.42;1.82) | 0.41 (0.19;0.86)* | 0.84 (0.36;1.97) | 0.39 (0.18;0.84)* |
| $\Delta$Parenting concerns ^d^ | | 1.19 (1.06;1.34)** | 1.09 (0.98;1.21) | 1.26 (1.10;1.45)** | 1.08 (0.95;1.24) |
| $\Delta$Parental satisfaction relationship child ^d^ | | 1.25 (0.78; 1.34) | 1.65 (0.88;1.67) |  |  |

^a^ Univariate regression analyses with factors at T1 and as covariate care use or the intensity of care use, i.e. number of contacts at T2; ^b^ Backward stepwise regression analyses were conducted with the difference score of the factor, if available, and care use at T1 as covariate. The criteria for taking a factor out of the model was set at P-value > 0.05. ^c^ The factors chronical condition was the only factor taken out of the model . ^d^ These factors are constructed as difference-of-scale scores between T2-T1.^e^ This factor is constructed as difference of dichotomic scores between T2-T1. *p<0.05 **p<0.01 ***p<0.001.
