# Supplementary material for: Care use and its intensity in children with complex problems are related to varying child and family factors: A follow-up study
Source: PLoS One. 2020 May 6;15(5):e0231620. doi: 10.1371/journal.pone.0231620 (PMC7202640; doi:10.1371/journal.pone.0231620)
Supplement: S2 Appendix — (DOCX) [file pone.0231620.s002.docx]

**Appendix: Interview protocol for the project Oké in Den Haag**

PONE-D-19-15380

Care use and its intensity in children with complex problems are related to varying child and family factors: a follow-up study Mrs. Noortje Pannebakker

Good afternoon, this is <name> speaking. I’m calling you on behalf of the project Oké in Den Haag [Translated: Okay in the city of The Hague]. You agreed with the nurse or doctor of the preventive youth healthcare center we could call you. I’m looking for the father or mother of <name child> , born at <date>. Is she or he in?

Thank you for the opportunity to talk to you about the project Oké in Den Haag. You agreed with the nurse or doctor of the preventive youth healthcare center we could call you. The nurse or doctor has already mentioned our project and also gave you a flyer. Did you get this flyer? I would like to explain the goals of this project some more. Is this a good moment to do so?

Would you like to be called back by a research assistant in your native language? We for example have Berber, Arabic, Turkish or English colleagues. Please tell me what is most convenient for you?

We would like to hear your opinion about the wellbeing of your family and child and if your families receives any treatment or you don’t have a need for care. Furthermore, if you get treatment, how are your experiences? We use your information to improve the psychosocial care for families in our city.

The project Oké in Den Haag is executed by the preventive youth healthcare services in The Hague [ad. In the Dutch protocol we named the organizations who deliver these services: Jong Florence and GGD], and other professional care providers.

We’re including around one thousand families from the city of The Hague. Parents are asked to fill in a questionnaire two times in the coming year. The first time is in the coming weeks, the second time one year later. We will not approach you for other activities.

The questionnaire is about the development and the upbringing of your child, your family and treatment you might or might not receive. You get an email with a secure link to the questionnaire. We expect it will take an hour to full in the questionnaire. Your answers will be treated confidential. This means that your data will be anonymous and that the professionals at the preventive youth healthcare services will not see the answers you gave. If you find filling in the questionnaire challenging, for example because of a language-barrier, we can support you. Please, let me know what is convenient for you.

To show our gratitude for your time and effort, we will sent you a digital gift card of 10 Euro when you finish the first questionnaire and a gift card of 25 Euro when you finish the second questionnaire.

Are you prepared to participate in this project and help to improve psychosocial care for children and families in the city of The Hague?

**If the respondent wants to participate:**

Thank you for participating in this project! You will receive an email with a link to the questionnaire. If you have any questions, you can reach us at <phone number> or <email address>. Are you prepared to complete the questionnaire within two weeks after receiving the email?

**If the respondent doesn’t want to participate:**

That’s too bad, but thank you for your time! Would you mind telling me why you decide not to participate?

- No time
- Not interested
- I don’t want to share my information
- Language barrier
- Other reasons
- No reason mentioned
