# Supplementary material for: Care use and its intensity in children with complex problems are related to varying child and family factors: A follow-up study
Source: PLoS One. 2020 May 6;15(5):e0231620. doi: 10.1371/journal.pone.0231620 (PMC7202640; doi:10.1371/journal.pone.0231620)
Supplement: S1 File — (PDF) [file pone.0231620.s003.pdf]

Medisch-Ethische Toetsingscommissie
Leiden | Den Haag | Delft

commissie METC-LDD
postzone P5-P
Mw. P.A. Visser

telefoon (071) 52 63241 of (071) 526 6963
e-mail metc-ldd@lumc.nl

onze referentie **C12.041**/PV/pv

uw referentie

datum 16 augustus 2019

onderwerp **Declaration 'No ethical review needed'**

aan De weledelzeergeleerde vrouwe
dr. M. Kamphuis

adres TNO child health
Schipholweg 77-89
2316 ZL LEIDEN

Dear Ms. Kamphuis,

The accredited medical research ethics committee (MREC) Leiden Den Haag Delft has received the proposal "1 gezin 1 plan, onderzoek naar de verbetering van zorgcoördinatie", registered under numer C12.041.

Documents received:
A1 Cover letter to MREC
C1 Research project proposal
C1 Summary of research project
F1 Questionnaire 'OKé in Den Haag'

Expedited review MREC
As no persons are subjected to procedures or are required to follow rules of behaviour, abovementioned research does not require a full review by the MREC under the Dutch Medical Research Involving Human Subjects Act (Dutch abbreviation: WMO).

It is your responsibility to ensure the research is carried out in accordance with the Dutch Medical Treatment Contracts Act (Dutch abbreviation: WGBO) and the General Data Protection Regulation (GDPR).

Yours sincerely,
on behalf of the MREC Leiden Den Haag Delft,

Mw. P.A. Visser
Secretary
