# Supplementary material for: Care use and its intensity in children with complex problems are related to varying child and family factors: A follow-up study
Source: PLoS One. 2020 May 6;15(5):e0231620. doi: 10.1371/journal.pone.0231620 (PMC7202640; doi:10.1371/journal.pone.0231620)
Supplement: S2 File — (DOCX) [file pone.0231620.s004.docx]

Additional information about the informed consent procedure

PONE-D-19-15380

Care use and its intensity in children with complex problems are related to varying child and family factors: a follow-up study Mrs. Noortje Pannebakker

[COMMENT]

please further clarify your consent procedure (for minors and adults) .

[RESPONSE]

Our respondent group consists of only parents or other caregivers. Minors were not part of the respondent group.

[COMMENT]

Please upload the original approval document, and its full translation in English. Moreover, please further clarify your consent procedure. Please provide as supporting information file a blank consent form used in this study. Please also provide an English translation.

[RESPONSE]

We used a two step informed consent strategy:

1. The professional with direct contact with the parent

- The professional informs the potential respondent about the study and it’s goals. We developed instructions for professionals to inform potential respondents;
- The professional stresses the anonymity of participation and that the professional doesn’t know if the parent decides to participate;
- The professionals asks permission to pass along the telephone number, which inclusion criteria are met and the location of the health center to the research team;
- When parents give this permission, they receive a flyer with information about the study.

1. The research assistant calling the potential respondent
   - The research assistant calls the parent and again informs the respondent about the study and it’s goals using a protocol (see document *Informed consent*);
   - Parents give informed consent, by opening a personalized link to the questionnaire sent to the email address of the parent.

[COMMENT]

Finally, please ensure that your previously published work (Pannebakker, Noortje M., et al. "Services use by children and parents in multiproblem families." Children and Youth Services Review 84 (2018): 222-228) is adequately mentioned in the Introduction and Discussion sections.

[RESPONSE]

The previous published cross sectional study included a subset of the response group of the current manuscript. The same set of inclusion criteria apply in both studies, but there is a difference in the required number of criteria to enter the study. The group of ‘multiproblem families’ was defined as meeting at least three of the five inclusion criteria, whereas the group of ‘children with complex problems or at risk on developing them’ is defined as meeting at least two of the five inclusion criteria.

Besides care use, we are in the current manuscript interested in factors associated with changes in intensity of care use, using a follow-up design. Multiproblem families, at the high end of the spectrum of complex problems, are known for their stable high intensity of care use. Children with complex problems or at risk on developing them provide a wider range of changes in intensity of use, which makes them a study group suitable for answering the research question.

We added the following text to the introduction section (new text has been italicized):

*In a previously published article on the data of the same cohort study we examined psychosocial care use by children from multiproblem families using a cross-sectional design [12]. In the current study we examined changes in intensity of care use besides changes in care use at all, in a study group of children with more variation in complex problems, using a follow-up design.* The aim of this study is to identify the changes in the predisposing, enabling and need factors that are associated 1. with a higher likelihood of changes in use of care services and 2. with changes in the intensity of use. As well as use of care services in general, we covered a subset, psychosocial care.

We also added the following text to our study in the discussion section (new text has been italicized)::

We found that several changes in predisposing and need factors were associated with changes in care use and its intensity, both for care use in general and for the use of psychosocial services. For several factors this confirms previous findings [10, 30-35]. In an earlier study we *equally found that child’s age and parenting concerns impacted psychosocial care use, but additionally found that psychosocial problems affects care use in general and psychosocial care [12]. For this earlier study we used a cross sectional design and took a subset of the study population, which were children from multiproblem families, who are at the high end of the spectrum of complex problems*. The current study adds ALE on the factors impacting intensity of care use. Unexpectedly, we found a slight negative relative risk between change in ALE and change in the intensity of use of any care. This is explained by the relatively large group of children whose ALE decreased while they continued to use care (see Table 2). ALE are relevant to the whole care process, i.e. not only care use itself, but also to its intensity.

In this study, changes in two enabling factors, social support and parental care use, were not associated with changes in care use or its intensity. This contrasts with earlier findings [10,*12*, 33-36].
